# Concentrating Nitrogen Waste with Electrodialysis for Fertilizer Production

**DOI:** 10.1021/acs.estlett.4c00595

**Published:** 2024-11-05

**Authors:** Mohammed Tahmid, Hyuck Joo Choi, Sai Tarun Ganapavarapu, Joseph Scott, Marta C. Hatzell

**Affiliations:** † School of Chemical and Biomolecular Engineering, Georgia Institute of Technology, 311 Ferst Drive NW, Atlanta, Georgia 30332, United States; ‡ George W. Woodruff School of Mechanical Engineering, Georgia Institute of Technology, 770 Ferst Drive NW, Atlanta, Georgia 30332, United States

**Keywords:** Ammonia, fertilizer, nutrient recovery, electrolysis, membrane stripping, resource recovery

## Abstract

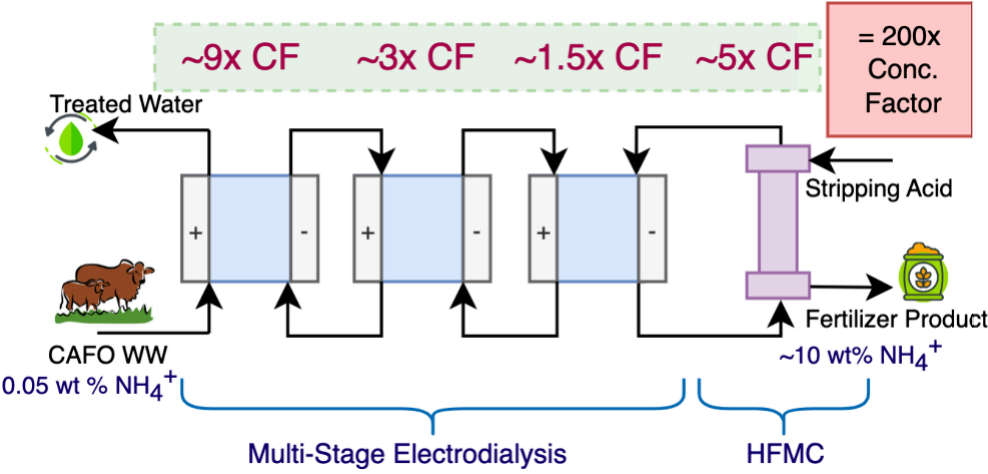

Recovery of nitrogen from wastewater presents a unique opportunity to valorize waste and contribute to a more circular nitrogen economy. However, dilute solution separations are challenging for most state-of-the-art separations technologies. This often results in technologies having low concentration factors that result in low-value products (e.g., < 1 wt % N). Here, we demonstrate how a cascading electrodialysis system combined with a hollow fiber membrane contactor (ED+HFMC) system can achieve efficient recovery of ammonia from simulated centralized animal feeding operation (CAFO) wastewater. The integrated system achieved an overall concentration factor of ∼200× (∼40× in ED and ∼5× in HFMC). This resulted in a ∼10 wt % NH_4_^+^-N fertilizer product. The specific energy consumption (SEC) for the three stages of the ED was 1.89–6.14 kWh/kg NH_4_^+^-N, which is lower than that of the Haber–Bosch process (8.9–19.3 kWh/kg N). Operating costs were <$0.90/kg NH_4_^+^-N for each of the electrodialysis stages and NH_3_ stripping. This integrated ED+HFMC system holds promise for the recovery of ammonia from dilute feedstreams as the ED+HFMC achieves high concentration factors and has low energy demand.

## Introduction

Today, 136 million tons of nitrogen-based fertilizers (NBF) are produced annually to meet agricultural needs.^1,2^ Of this nitrogen, about 25% ends up in agricultural wastewater.^3−5^ Recovery of nitrogen from agricultural waste streams provides a unique opportunity to valorize waste to meet agricultural needs (e.g., fertilizer production). A critical bottleneck in achieving this goal is the relatively dilute nature of nitrogen in wastewater. The typical mean nitrogen content is ∼206 mg-N/L for fertilizer production effluent, ∼1333 mg-N/L for CAFO wastewater, and ∼10 mg-N/L municipal wastewater effluent.^6^ Thus, from a performance perspective, any technology for nitrogen recovery must be able to achieve a high concentration factor (e.g., ∼40–200×) to convert wastewater nitrogen into a fertilizer grade product (e.g., 10 wt % N).^7^ The cost of fertilizers today is also low ($1.06–1.43/kg-N).^8^ Thus, from an economic perspective, any nitrogen recovery technology for fertilizer production must be able to achieve low-cost separations.

Several investigations have examined a variety of different technologies to achieve the separation of nitrates and ammonium from wastewater.^9^ The most common technology examined is selective ion exchange resins.^10^ Ion exchange resins use charge to selectively adsorb ammonium or nitrate from the waste mixture. The resin is then regenerated using a salt solution, and the ammonium and/or nitrate are released into a separate stream. The concentration factor for an ion-exchange resin is dependent on the ion exchange capacity of the resin, the mass of the resin, and the volume of the regeneration solution. Reports suggest that a concentration factor for an ion-exchange resin can be between ∼4 and 10× ,^11−13^ suggesting that the output nutrients may be 0.2–0.5 wt % N.^14,15^ The cost of recovering this nitrogen varies in the range of 2.53–3.01 USD/kg-N.^16,17^ Beyond ion exchange resins, others have investigated ammonia stripping,^18−21^ adsorption-based separations,^22,23^ and membrane-based separations.^11,12,24−26^ The concentration factors for these separations have approached 3–8× for ammonia stripping, 1.6–7× for adsorption-based separations, and 3–9× for membrane-based separations. Although most of these investigations did not optimize for the concentration factor, the examinations provide some insight into the limited concentration factor of each singular approach. Cost estimates for the recovery of nitrogen-based nutrients are not always reported, but some suggest that the cost of ammonia stripping, adsorption-based separation, and membrane-based separations may be $1.4–1.5,^18−21^ $2.53–3.01,^11−13^ and $0.1–1.4^11,12,24,25^ per kg-N. The energy demand for ammonia stripping, adsorption-based separation, and membrane-based separations has also been suggested to be 0.1–0.2,^18−21^ ∼0.1,^11−13^ 0.2–13^11,12,24,25^ kWh/kg-N.

More recently, with the growing focus on widespread electrification and electrochemical-based separations,^27,28^ investigations have examined nutrient recovery using electrodialysis, capacitive deionization, and battery-electrode-based deionization.^29−33^ Capacitive deionization and battery deionization have demonstrated selective ammonia recovery. The energy and concentration factors for these technologies are 0.45–12 kWh/kg N and 3–12×. Electrodialysis (ED) has been demonstrated for decades.^34,35^ Ammonia recovery has been demonstrated through both a standard configuration cell and a bioplar membrane electrodialytic cell. In the former configuration, ammonia is removed as ammonium, whereas in the bipolar membrane configuration, ammonium is stripped through a pH swing process. The coupling of gas-permeable membranes (GPM) with ED has been used for selective ammonia recovery. Elevating the pH to shift the NH_3_/NH_4_^+^ equilibrium toward NH_3_ allows only NH_3_ gas to diffuse across the GPM, while competing ions remain in solution. This pH elevation has been achieved either by using alkaline wastewater^36^ or by employing bipolar membrane ED^37^ to generate the base in situ. Hollow fiber membrane contactors^38^ have also been investigated for ammonia recovery from wastewater due to their high packing density and large total surface area,^39^ which enhance the mass transfer efficiency of the recovery process. Although these processes report high ammonia recovery (67–90%), hollow fiber membrane contactors are often examined using high-strength wastewater that is not dilute (NH_4_^+^-N = 1700–10,000 mg/L). Since many sources of wastewater have a much lower NH_4_^+^ content, achieving a significant concentration factor beyond ∼2× has proved challenging.

Logistical constraints and agricultural demands require a high nitrogen content in fertilizers (>2 wt % N for liquid organic, >2.5 wt % N for solid organic, >5 wt % N for liquid inorganic, and >10 wt % N for solid inorganic).^7^ Achieving these nitrogen levels requires separation techniques to achieve a very high concentration factor (∼40–200×). As single techniques are inadequate to achieve the required concentration factors, a combination of techniques or an integrated system is needed.

Wastewater in manure storage basins or anaerobic treatment lagoons in confined animal feeding operations (CAFO) has high levels of nitrogen, primarily in the NH_4_^+^ ion form.^40^ The composition of CAFO wastewater (WW) varies significantly depending on the location of the facility, type of animals (e.g., cattle, swine, poultry), and farm management practices. The NH_4_^+^-N concentration has been reported to be in the range of 180 to 3540 mg/L for swine and 10 to 510 mg/L for cattle feedlots.^41^ Two types of model wastewater, simplified CAFO WW and synthetic CAFO WW, have been used to demonstrate nitrogen recovery in this study. Both of these model wastewaters contain ∼500 mg/L of NH_4_^+^-N. This concentration falls within the reported range for NH_4_^+^-N in swine and cattle feedlot wastewater, ensuring that the conditions are representative of typical CAFO wastewater.^41^ The use of synthetic wastewater allows for controlled and consistent experimental conditions while avoiding the variability in composition associated with real wastewater samples, thus improving the reproducibility and applicability of the findings.

Here, we focus on concentrating nitrogen waste with integrated electrodialysis coupled to a hollow fiber membrane contactor (ED+HFMC) system for highly concentrated NBF production. We focus on detailing the concentration factor that can be achieved using staged electrodialysis (ED). Next, we study the integration of cascading ED with a HFMC, which strips NH_3_ from wastewater using an acid. The final achievable concentration factor and nitrogen content are determined for the integrated system, and the proposed approach is compared with the conventional Haber–Bosch process for ammonia production in terms of energy consumption, GHG emissions, and cost. Finally, the potential of an integrated ED+HFMC system for producing NBFs in large-scale continuous applications is discussed.

## Methods and Materials

### Synthetic CAFO Wastewater

Analytical grade reagents and DI water were used to prepare the simplified CAFO WW, synthetic CAFO WW, and 0.4 M Na_2_SO_4_ electrode rinse solution for the ED experiments. The simplified CAFO WW contains a typical NH_4_^+^-N concentration found in real CAFO WW, while the synthetic CAFO WW mimics the overall composition of the real CAFO WW, including representative species other than NH_4_^+^-N (recipe in Supporting Information Table S1). In addition, stock solutions were used to prepare 0.4 M sulfuric acid and 50% sodium hydroxide for the HFMC experiments.

### Electrodialysis For Concentrating Nitrogen Waste

Batch electrodialysis experiments were conducted in an ED 64002 bench-scale electrodialyzer (PCCell GmbH, Germany) in chronopotentiometry mode (constant current applied using a BioLogic VMP3 multichannel potentiostat). The ED cell consists of a Pt/Ir-coated titanium anode, a stainless steel cathode, five cell pairs (active area of 8 × 8 cm^2^ per membrane) and spacers. Each cell pair consists of one anion exchange membrane (PC SA, PCCell GmbH, Germany) and one cation exchange membrane (PC SK, PCCell GmbH, Germany). The voltage and electrical conductivities were monitored continuously. In ED, application of an electric potential drives the transport of ions through ion-exchange membranes, resulting in the concentration of one stream (concentrate) and the dilution of another (diluate).^42^ The diluate and concentrate streams were sampled at 1 h intervals, and ion concentrations (NH_4_^+^) were measured using cation chromatography (Dionex Aquion, IonPac CS17 column).

The specific energy consumption (SEC, kWh/kg NH_4_^+^-N) of the ED stages is defined as the electrical energy consumed to recover a unit mass of NH_4_^+^-N and is calculated with experimentally recorded voltage data (*U*(*t*)). The energy consumption for pumping is assumed to be negligible and is thus not included in the equation:^37^
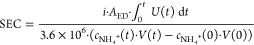
1where *i* is the applied current density (A m^–2^), *A*_ED_ is the effective membrane area (m^2^) of the ED unit, *t* is the ED unit operation duration (s), and *c*_NH_4__^+^(0) and *c*_NH_4__^+^(*t*) are the initial and final NH_4_^+^ concentrations (kg L^–1^) in the concentrate streams, while *V*(0) and *V*(*t*) are the initial and final volumes (L) of the concentrate.

The following equation was used for estimating the maximum achievable concentrate concentration in ED (*C*_max_; i.e., in the absence of any osmosis effects):
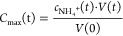
2

### Hollow Fiber Membrane Contactor (HFMC) for Further Concentration and Selective Ammonia Recovery

A commercial HFMC (3M Liqui-Cel EXF-2.5 × 8) with a shell-lumen configuration and an active membrane area of 1.4 m^2^ (polypropylene X50 fiber) was used to strip ammonia from ED concentrated synthetic CAFO WW. The synthetic CAFO WW was basified using 50% NaOH to a pH greater than 11 before being pumped through the shell side of the device while the acid stripping solution (H_2_SO_4_ used in this study) was counter-currently circulated through the lumen side. Ammonia is in gas form at pH > 9.25 (p*K*_a_), thus it diffuses from the feedwater across the membrane to react with the acid stripping solution in the lumen side producing ammonium sulfate.^43^ The feed synthetic CAFO WW and acid stripping solutions were sampled at 1 h intervals, and ion concentrations (NH_4_^+^, Na^+^ and K^+^) were measured using cation chromatography (Dionex Aquion, IonPac CS17 column). The pH of the acid stripping solution was monitored continuously (Thermo Orion Star A215 meter with ROSS Ultra pH triode), and small amounts of H_2_SO_4_ were added when the pH increased above 2 to maintain the driving force for ammonia transfer. Details of operating conditions used for both ED and HFMC experiments are provided in Supporting Information Table S2.

## Results and Discussion

### Concentrating Ammonia with a Staged Electrodialysis System

**Figure 1:**
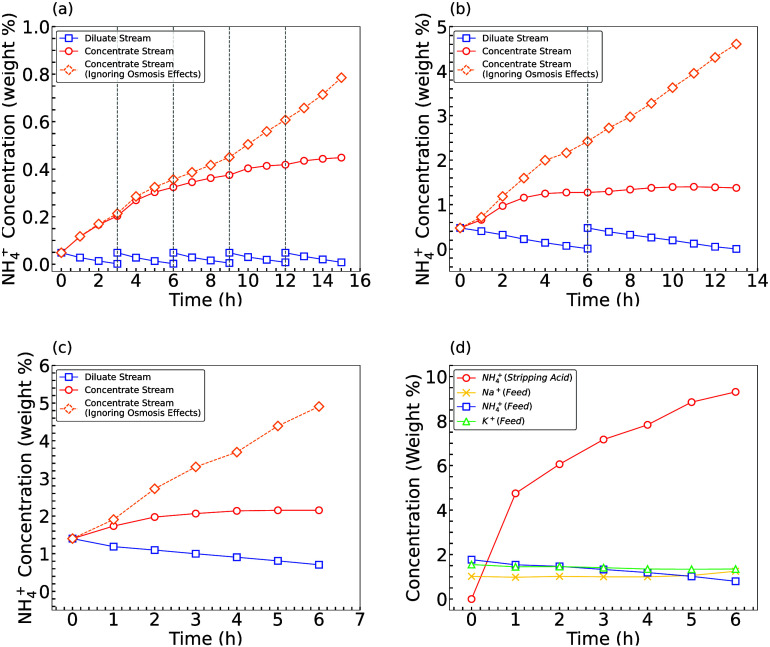
NH_4_^+^-N concentration variation with time for (a) stage 1 ED, (b) stage 2 ED, (c) stage 3 ED (gray dashed line shows when a diluate batch was replaced), and (d) NH_4_^+^-N and competitor ion concentration variation with time for stripping acid and feedwater in HFMC experiments.

ED was performed in stages to determine the maximum NH_4_^+^-N concentration factor obtainable and the maximum exit concentration. Simplified CAFO WW (∼500 ppm/0.05 wt % NH_4_^+^-N) was used as both diluate and concentrate feed for stage 1 ED. A 0.45 wt % NH_4_^+^-N water concentrate was achieved after 15 h, resulting in a ∼ 9× concentration factor (Figure 1a). The diluate stream was replaced when the conductivity dropped below 1 mS/cm to ensure sufficient NH_4_^+^-N availability and to prevent voltage escalation due to the low conductivity of the diluate stream (Figure S4). After four replacements of the diluate feedstream, the NH_4_^+^-N concentration in the concentrate stream plateaued due to an increase in the osmotic flux of water from the diluate to the concentrate. This was indicated by an increase in the volume of the concentrate solution. Using eq 2, we show that the maximum achievable concentration in the absence of osmosis effects is ∼0.8 wt % NH_4_^+^-N.

In the second stage of ED, the initial feed concentrations of the diluate and concentrate streams were set to the exit of stage 1 (e.g., 0.45 wt % NH_4_^+^-N). This yielded a 1.37 wt % NH_4_^+^-N water concentrate. The second stage of the ED exhibited more pronounced osmosis effects and a much lower concentration factor (∼3×; Figure 1b). In the third stage of ED, the initial feed concentration of the diluate and concentrate was established at the end of stage 2 (e.g., 1.37 wt % NH_4_^+^-N). This yielded a 2.15 wt % NH_4_^+^-N water concentrate. As anticipated, the third stage exhibited even more pronounced osmosis and the lowest single stage concentration factor of ∼1.5×. The maximum achievable concentration without osmosis effects would have been ∼5 wt % NH_4_^+^-N (Figure 1c).

### Concentrating Ammonia with a Staged Electrodialysis in Series with a Hollow Fiber Membrane Contactor

**Figure 2:**
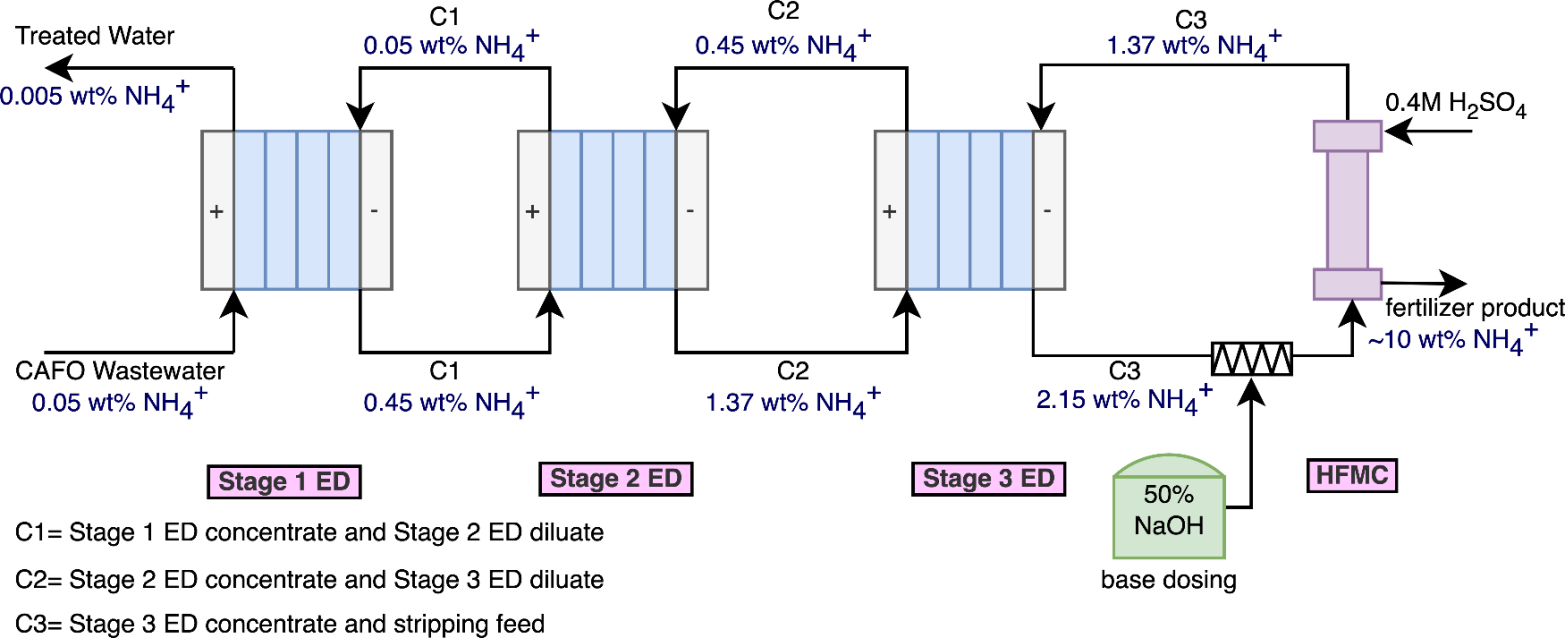
Scheme for large-scale continuous process to produce highly concentrated NBF.

ED alone produced a maximum concentration of only 2.15 wt % NH_4_^+^-N concentrated product. Above this concentration, osmosis dominates transport through the ion-exchange membranes. This was not surprising, as the ion exchange capacity of the membranes is limited to 2–3 M. Thus, to obtain higher concentrations, we next coupled the effluent of the third ED stage with a HFMC. A ∼40× concentrated synthetic CAFO WW (∼2 wt % NH_4_^+^-N), basified to pH > 11, was used as feed to the HFMC, and 0.4 M sulfuric acid was used as the stripping solution. A ∼10 wt % NH_4_^+^-N fertilizer product was produced after 6 h of operation (Figure 1d). Competitor cations (e.g., Na^+^, K^+^) were present in the feed but were not detected in the acid stripping solution, indicating a selective recovery of ammonia. This suggests that an integrated cascading ED + HFMC system would be suitable for large-scale continuous process applications to produce highly concentrated NBFs (Figure 2).

**Figure 3:**
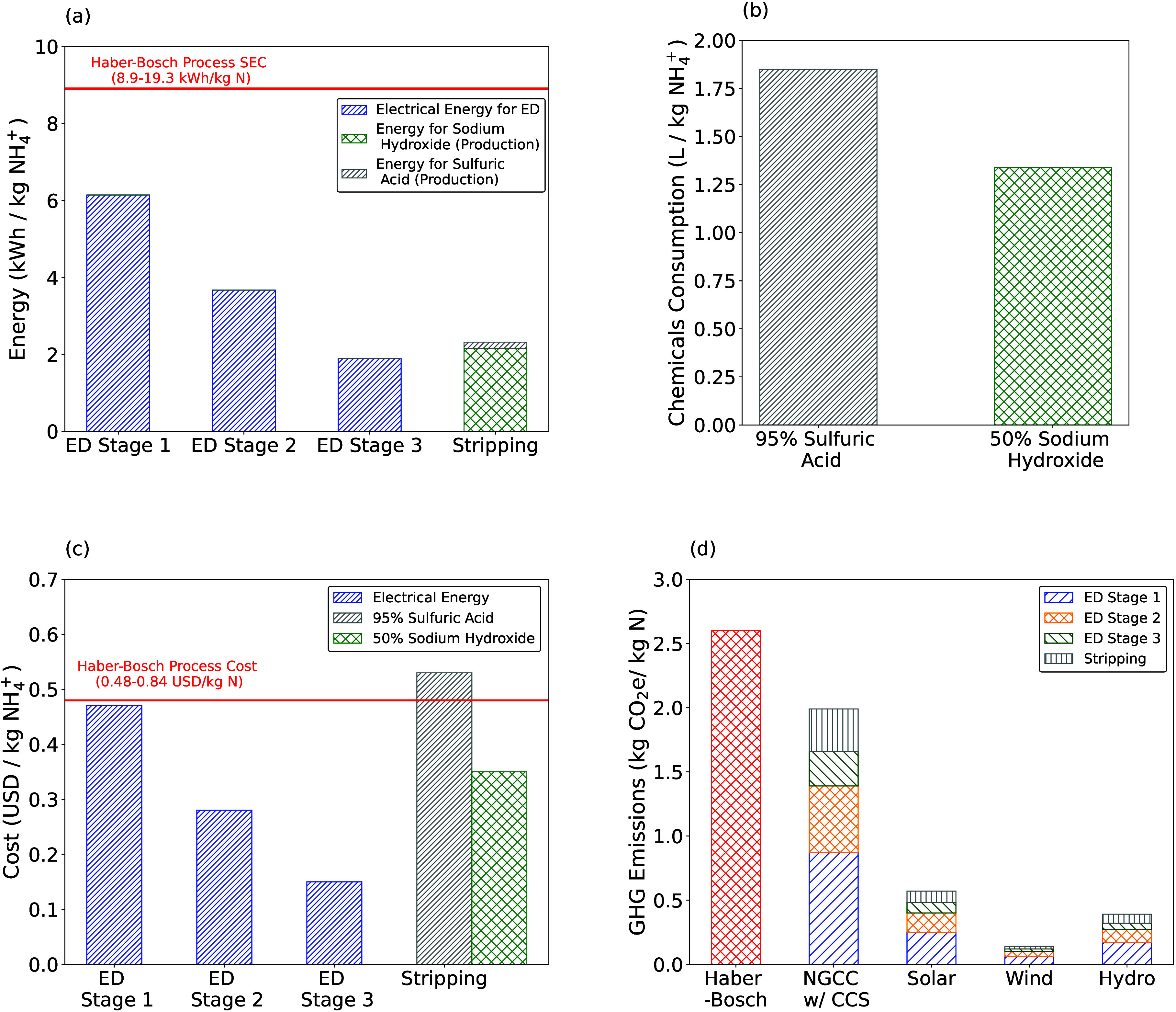
(a) Electrical energy consumption for ED stages and stripping. Electricity has been directly applied for ED, while the energy demand for sulfuric acid and sodium hydroxide production (∼50 kWh/MT sulfuric acid^48^ and ∼2120 kWh/MT sodium hydroxide^49^), which indirectly affects the overall energy demand for this process, has been used to determine the energy demand of using these chemicals per kg NH_4_^+^-N produced. (b) Chemicals consumption per kilogram of NH_4_^+^-N produced in stripping. (c) Costs per kilogram of NH_4_^+^-N produced for each ED stage and stripping, and (d) GHG emissions of powering the process with nonrenewable fuel (Natural Gas Combined Cycle with Carbon Capture and Sequestration, NGCC w/CCS), various renewable sources (solar, wind, and hydro) vs GHG emissions of the Haber–Bosch process per kilogram of NH_4_^+^-N produced.

The integrated cascading ED+HFMC process was found to be efficient in producing concentrated NBF (∼10 wt % NH_4_^+^-N) from nitrogen waste. An overall concentration factor of ∼200× (from ∼0.05 wt % NH_4_^+^-N in CAFO WW to ∼10 wt % NH_4_^+^-N fertilizer product) was achieved, while other comparable studies have reported a maximum concentration factor of ∼2.5× (Table S5). The SEC in the three ED stages (1.89–6.14 kWh/kg NH_4_^+^-N; Figure 3a) were found to be much lower than the Haber–Bosch process (8.9–19.3 kWh/kg N).^37^ The operating costs for the proposed process are <$0.90/ kg NH_4_^+^-N for each of the ED stages and stripping (Figure 3c), which is comparable to or lower than the Haber–Bosch process ($0.48–0.84/kg N).^44^ Furthermore, the use of renewable sources to generate electricity could reduce GHG emissions by up to 95% compared with the Haber–Bosch process (Figure 3d). Pilot plant studies could help identify optimal conditions to improve process efficiency, potentially reducing ammonia production costs through economies of scale in large-scale plants. However, a key challenge arises during the later stages of the ED process: as the diluate stream becomes increasingly dilute over time, the ohmic resistance for ion transport increases, leading to an increase in the voltage of the ED cell (Figure S4). This increased voltage negatively affects the energy demand for the process. To address this high resistance, integration of this system with other electrochemical techniques, such as redox-coupled reactions and electrodeionization (EDI), offers potential solutions. For example, the hydroquinone (HQ) and benzoquinone (BQ) redox couple has been explored due to its theoretical redox potential of 0 V, significantly lower than the 1.2 V required for water electrolysis in ED,^45^ presenting opportunities for further energy reductions for ammonia separations. However, the HQ/BQ system is limited by the low solubility and stability at pH > 9, which limits the current density that can be applied.^45^ Another promising approach to reduce ohmic resistance in dilute streams is the use of EDI, which incorporates ion exchange membranes and resin particles.^46^ Using porous materials in the feed channels, EDI creates low-resistance pathways for ions that improve energy efficiency, particularly for dilute stream separations.^47^ Both of these methods could reduce the ohmic resistances in the dilute process streams, improving the energy efficiency for the recovery of ammonia from wastewater. Here, we demonstrate the potential of a cascading ED+HFMC process for the recovery of ammonia from simulated wastewater. Moving toward implementation will require evaluation of the system performance for large-scale systems operating continuously with real wastewater. Here, we demonstrate that synergies can exist when coupling multiple separation technologies together, to get concentration factors which exceed 200×. Specifically, we find that ED is ideal for concentrating dilute concentrations, whereas the hollow fiber membrane contactor is effective at concentrations in the midrange to high concentrations. We also examined the cost of these systems, and found that the capital costs of ED, are expected to be similar to those of brine desalination systems, with estimates of around $2.8 million for a 3000 m^2^ membrane area system with a scaling factor of 0.7 for membrane areas between 1500 and 7000 m^2^.^50^

## Supporting Information Available

Equations for the calculation of chemical consumption, cost, current efficiency, ammonia flux, GHG emissions; simplified and synthetic CAFO wastewater recipe; operating conditions for ED and NH_3_ stripping experiments; basis for cost analysis; life cycle GHG emissions for power generation; comparison of performance metrics of comparable studies; photo of ED and HFMC system setup; mechanism of HFMC operation and dissociation equilibrium between NH_3_ and NH_4_^+^; voltage data for ED; pH data for HFMC; ammonia flux and current efficiency (PDF)
