# Supplementary material for: Concentrating Nitrogen Waste with Electrodialysis for Fertilizer Production
Source: Environ Sci Technol Lett. 2024 Nov 5;11(12):1413–9. doi: 10.1021/acs.estlett.4c00595 (PMC11636197; doi:10.1021/acs.estlett.4c00595)
Supplement: Supplementary file 1 — ez4c00595_si_001.pdf [file ez4c00595_si_001.pdf]

Supporting Information: Concentrating nitrogen waste with electrodialysis for fertilizer production

Mohammed Tahmid ,[†] Hyuck Joo Choi,[†] Sai Ganapavarapu,[†] Joseph Scott,[†] and Marta C. Hatzell[*,‡,†]

†*School of Chemical and Biomolecular Engineering, Georgia Institute of Technology, 311 Ferst Drive NW, Atlanta, GA, 30332, United States*

‡*George W. Woodruff School of Mechanical Engineering, Georgia Institute of Technology, 770 Ferst Drive NW, Atlanta, GA, 30332, United States*

E-mail: marta.hatzell@me.gatech.edu

Phone: +123 (0)123 4445556. Fax: +123 (0)123 4445557

Equations

Chemicals Consumption

$$H_2SO_4 \text{ consumption} \left(\frac{\text{L } 95\% \text{ } H_2SO_4}{\text{kg } NH_4^+}\right) = \frac{H_2SO_4 \text{ used for stripping (mL)}}{NH_4^+ \text{ produced in stripping (kg)} \times 1000} \quad \text{(S1)}$$

where 200 mL 0.4 M H_2SO_4 ($\sim$4.489 mL of 95% H_2SO_4) was initially used as the stripping solution, and a total of 30 mL 95% H_2SO_4 was added subsequently to maintain pH < 2, while 18.62 g of NH_4^+ was recovered in the stripping.

$$\text{NaOH consumption} \left(\frac{\text{L } 50\% \text{ NaOH}}{\text{kg } NH_4^+}\right) = \frac{\text{NaOH used for stripping (mL)}}{NH_4^+ \text{ produced in stripping (kg)} \times 1000} \quad \text{(S2)}$$

where 25 mL of 50% NaOH was used to basify 1L of synthetic CAFO WW above pH>11

Cost Calculation

$$\text{Electricity cost}\left(\frac{\text{USD}}{\text{kg NH}_4^+}\right) = \text{Electricity consumed}\left(\frac{\text{kWh}}{\text{kg NH}_4^+}\right) \times \text{Unit cost}\left(\frac{¢}{\text{kWh}}\right) \tag{S3}$$

$$\text{H}_2\text{SO}_4\ \text{cost}\left(\frac{\text{USD}}{\text{kg NH}_4^+}\right) = \frac{V_{\text{acid}} \times C_{\text{acid}} \times n_{\text{acid}} \times M_{\text{acid}}}{10^6} \tag{S4}$$

where V_{acid} is the volume of acid consumed (L) per kg NH_4^+ produced, C_{acid} is the unit cost of acid (USD/MT), n_{acid} is the molar mass of acid (g/mol), and M_{acid} is the molarity of acid (mol/L).

$$\text{NaOH cost}\left(\frac{\text{USD}}{\text{kg NH}_4^+}\right) = \frac{V_{\text{base}} \times C_{\text{base}} \times n_{\text{base}} \times M_{\text{base}}}{10^6} \tag{S5}$$

where V_{base} is the volume of base consumed (L) per kg NH_4^+ produced, C_{base} is the unit cost of base (USD/MT), n_{base} is the molar mass of base (g/mol), and M_{base} is the molarity of base (mol/L).

Unit costs for electricity, acid and base used for calculations are provided in Table S3.

Current Efficiency

The current efficiency for NH_4^+, $C_{NH_4^+}$ (%) measures the effective utilization of current for transporting NH_4^+ ions. It is defined as the ratio of the amount of electrical charge transported by NH_4^+ ions to the total electrical charge applied. This efficiency can be estimated from the change in NH_4^+ concentration in the diluate as follows:[1]

$$C_{NH_4^+}(\%) = \left(\frac{zF\Delta n}{NQ}\right) \times 100 \tag{S6}$$

with

$$Q = \int_0^t I\, dt \tag{S7}$$

where z is the charge of the ion transported, F is the Faraday constant (96485 As/mol), Δn denotes the change in the amount of NH_4^+ in the diluate (mol), N is the number of cell pairs in the ED stack (5 in this study), Q is the amount of electricity passed during

time t in the ED process (A.s) and I is the current (A).

Ammonia Flux

The flux of NH_4^+, $J_{NH_4^+}$ (g NH_4^+ m^{-2} h^{-1}) through the ion exchange membranes in Electrodialysis is given by:

$$J_{NH_4^+} = \frac{\Delta C_{NH_4^+} \times V_{dil}}{NA\Delta t} \tag{S8}$$

where $\Delta C_{NH_4^+}$ is the change in concentration of NH_4^+ in the diluate (g/L), V_{dil} is the volume of the diluate (L), A is the area of one ion exchange membrane (m^2), and Δt is the time interval (h).

GHG Emissions

Greenhouse gas (GHG) emissions per kg NH_4^+ produced has been calculated based on life cycle GHG emissions data (Table S4) for various renewable and non-renewable power sources using:

$$\text{GHG emission}\left(\frac{\text{kg CO}_2\text{e}}{\text{kg N}}\right) = \frac{\text{Electricity}\left(\frac{\text{kWh}}{\text{kg NH}_4^+}\right) \times \text{GHG per kWh}\left(\frac{\text{g CO}_2\text{e}}{\text{kWh}}\right)}{1000} \tag{S9}$$

Tables

Table S1: Simplified and Synthetic CAFO wastewater recipe in 1L DI water

**Component**	**Simplified CAFO Wastewater (mg)**	**Synthetic CAFO Wastewater (mg)**
NH_4Cl	1557	500
Na_2HPO_4	-	672
Urea	-	168
$(NH_4)_2CO_3$	-	950
K_2CO_3	-	1073

Table S2: Operating conditions for ED and NH_3 stripping

Condition	ED Stage 1	ED Stage 2	ED Stage 3	Stripping
Flow rate (L/h)	5	5	5	10
Current density (ED) (mA/cm^2)	0.75	3.75	6.00	-
Number of cell pairs (ED) (AEM+CEM)	5	5	5	-
Volume of diluate (ED) (mL)	500	500	500	-
Volume of concentrate (ED) (mL)	100	100	100	-
Number of diluate batch replacements (ED)	4	1	None	-
Volume of feed wastewater (stripping) (mL)	-	-	-	1000
Volume of stripping acid (stripping) (mL)	-	-	-	200

Table S3: Basis for cost analysis

	Cost	Unit	Comment	Reference
Electricity	7.73	¢/kWh	US average for industry	[2]
Sulfuric acid	162.5	$/MT	95% (bulk)	[3]
Sodium hydroxide	350	$/MT	Solid (bulk)	[4]

Table S4: Life Cycle GHG Emissions for Power Generation

Power Generation Source	Life Cycle GHG Emissions (gCO_2e/kWh)[5]
NGCC w/ CCS [a]	142.3
Solar	40
Wind	10
Hydro	28.2

[a] *Natural Gas Combined Cycle (NGCC) with carbon capture and sequestration (CCS)*

Table S5: Comparison of performance metrics of studies that have coupled electrochemical cell with gas permeable membrane (GPM) to recover and concentrate ammonia from wastewater

**Method**	**Electrochemical cell configuration**	**GPM geometry**	**Feed NH_4^+ concentration (ppm)**	**Current Density ($A\ m^{-2}$)**	**Concentration Factor for NH_4^+**	**Nitrogen Flux ($g\ N\ m^{-2}\ d^{-1}$)**	**Energy Consumption ($kWh\ kg^{-1}\ N$)**	**Reference**
This study	ED with 5 cell pairs	Hollow fiber	524	7.5-60	200	60-456	1.89-6.14	
Electrochemical Stripping	Single CEM	Flat sheet	7490	100	0.3	1010	8.5	[6]
Electrochemical stripping	Single CEM	Flat sheet	30-3000	100	0.8	13.4	N/A	[7]
Electrochemical Cell+ TMCS	Single CEM	Tubular	2562	50	N/A	335	13.6	[8]
LLMC + ED	ED with 5 cell pairs	Hollow fiber	1700-4000	Variable (constant voltage = 7V)	1.5-2.2	538-1920	0.9-8.9	[9]
BMED + GPM	4-chamber cell with 1 AEM, 1 CEM, 1 BPM	Flat sheet	3360-3772	200	2.5	953	25.8	[10]
BMED + HFMC	ED with BPM	Hollow fiber	1750	100	N/A	819	5.1	[11]
Proton-Mediated Redox Couple + GPM	3-channel cell with 2 CEM	Flat sheet	90	9.6	0.4	12.4	12.3	[12]

ED: Electrodialysis, BMED: Bipolar Membrane Electrodialysis, TMCS: TransMembrane ChemiSorption, LLMC: Liquid-Liquid Membrane Contactor, GPM: Gas Permeable Membrane, HFMC:, Hollow Fiber Membrane Contactor, AEM: Anion Exchange Membrane, CEM: Cation Exchange Membrane, BPM: Bipolar Membrane, N/A: Not Available

Figures

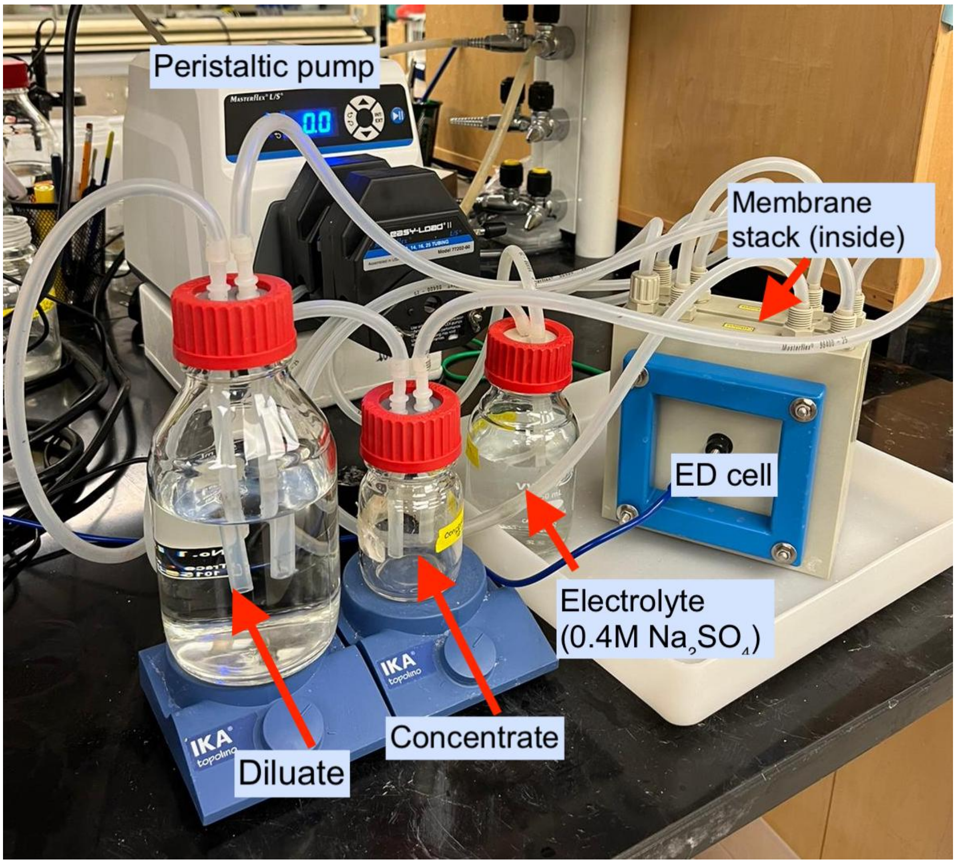

Figure S1: ED system set up in the lab

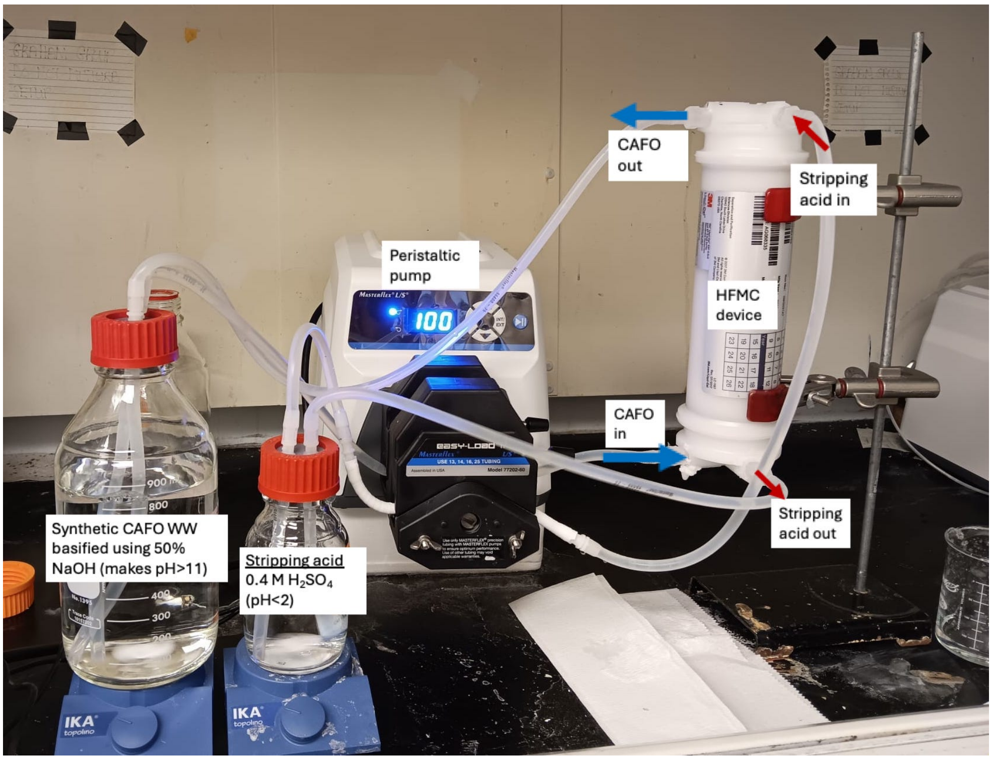

Figure S2: HFMC (NH_3 stripping) system set up in the lab

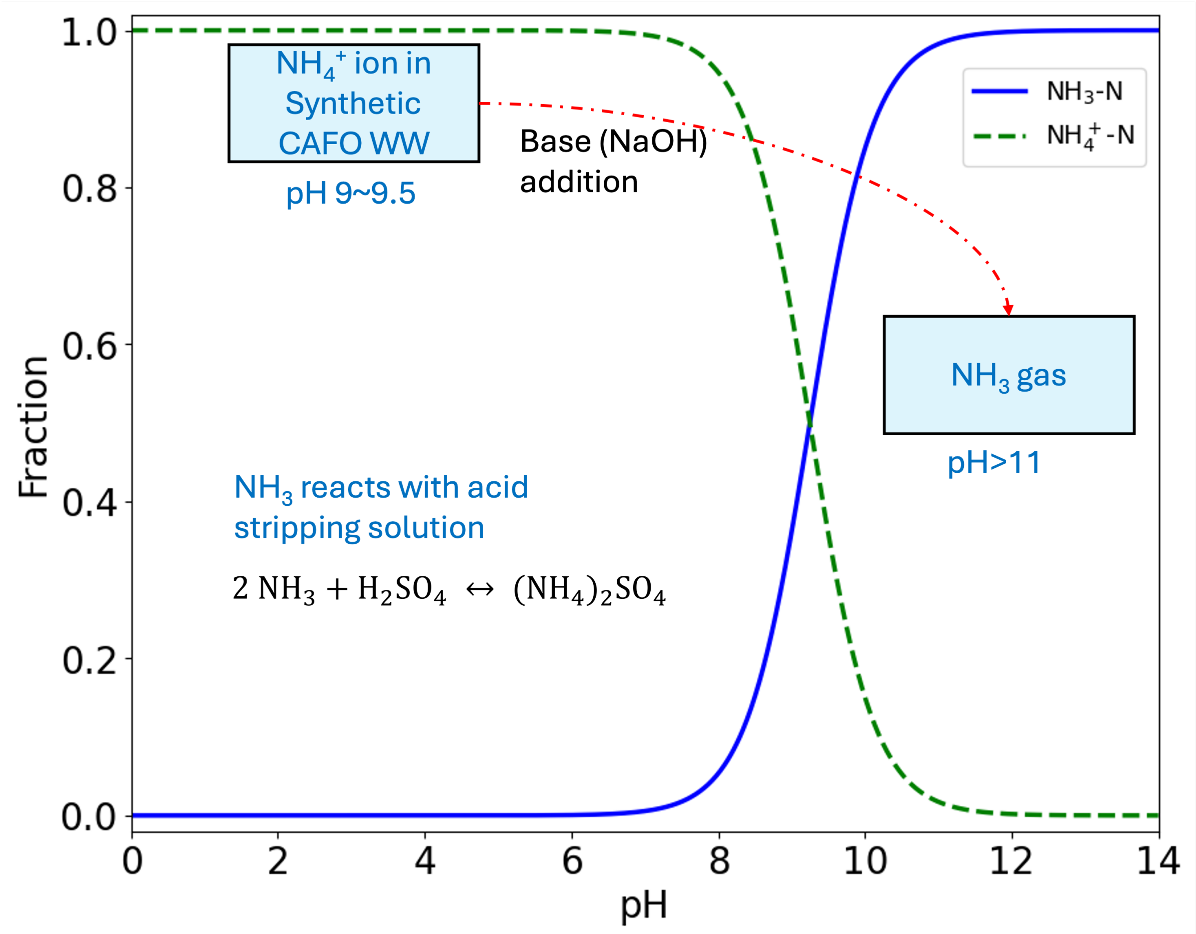

Figure S3: Mechanism of HFMC operation and dissociation equilibrium between ammonia, NH_3, and ammonium, NH_4^+, as a function of pH. Ammonium has a pKa of 9.25 at 25 °C.

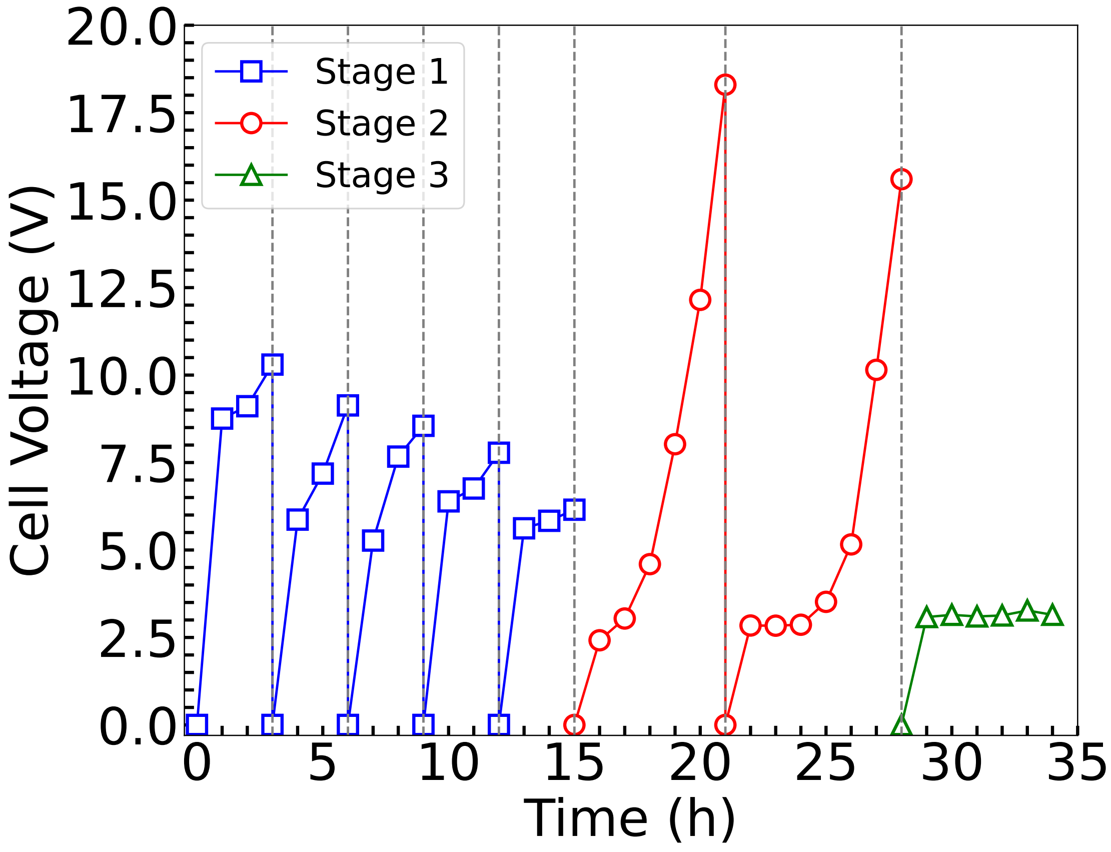

Figure S4: Voltage data for Electrodialysis. Gray dashed line shows when a diluate batch was replaced

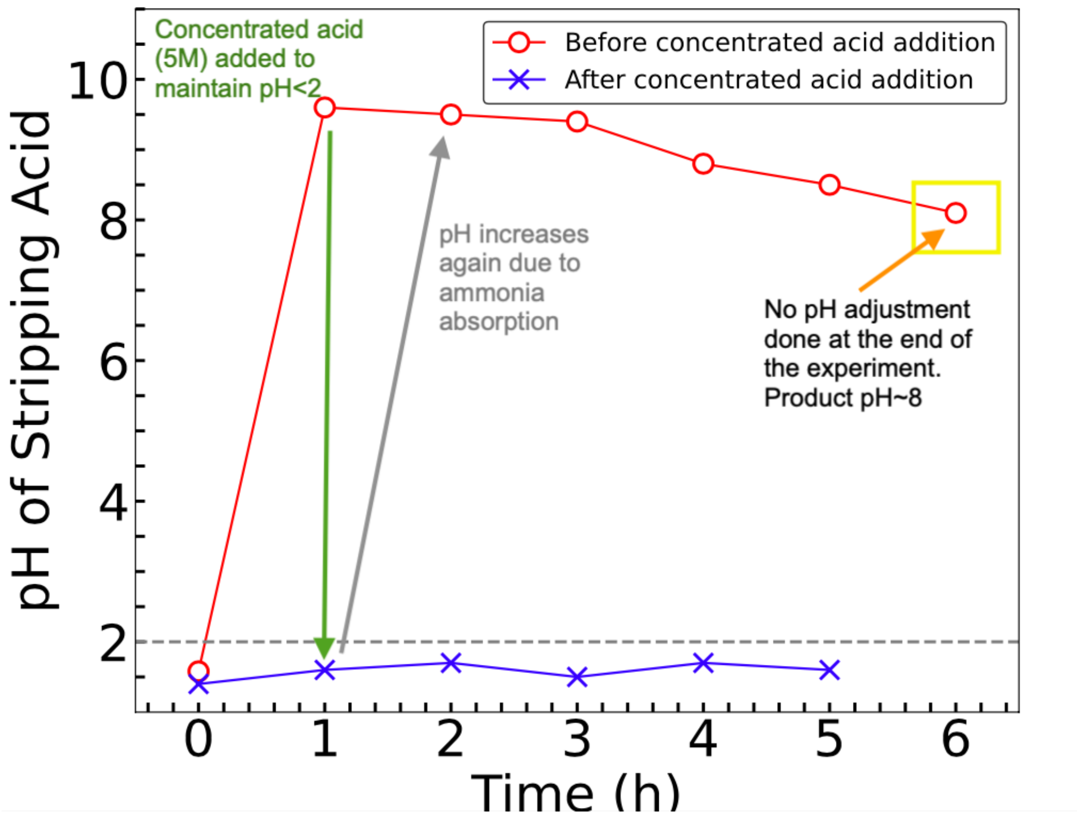

Figure S5: pH of stripping acid solution before and after concentrated acid addition. Concentrated acid was added every hour when pH measurements were done to re-adjust the stripping solution pH to <2

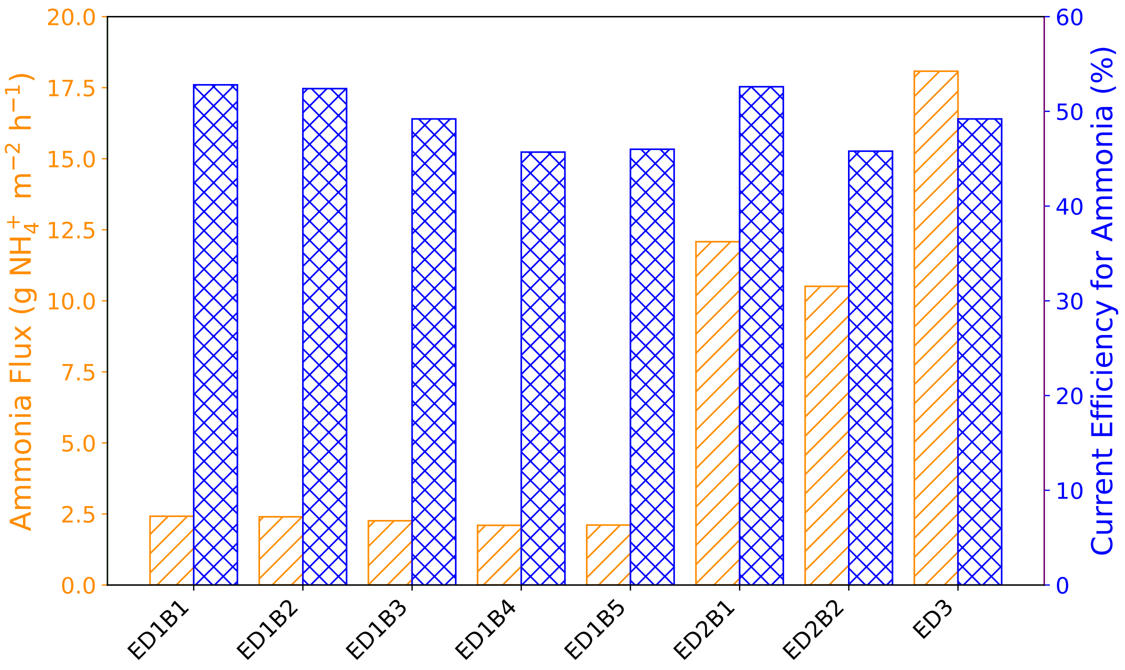

Figure S6: Ammonia flux though the ion-exchange membranes in electrodialysis and current efficiency for ammonium ion transfer. The notations x and y in EDxBy represent the ED stage and diluate batch respectively

References

(1) Nagarale, R.; Gohil, G.; Shahi, V. K.; Trivedi, G.; Rangarajan, R. Preparation and electrochemical characterization of cation-and anion-exchange/polyaniline composite membranes. *Journal of Colloid and Interface Science* **2004**, *277*, 162–171.

(2) U.S. Energy Information Administration Electric Power Monthly: Table 5.6.A. Average Price of Electricity to Ultimate Customers by End-Use Sector, by State, May 2024 and 2023. 2024; https://www.eia.gov/electricity/monthly/epm_table_grapher.php?t=epmt_5_6_a, Accessed: 2024-07-05.

(3) Producer Price Index for Crude Petroleum. https://fred.stlouisfed.org/series/WPU0613020T1, 2024; Accessed: July 5, 2024.

(4) Business Analytiq Caustic Soda Price Index. https://businessanalytiq.com/procurementanalytics/index/caustic-soda-price-index/, 2024; Accessed: July 5, 2024.

(5) What are the greenhouse gas emissions of a mini-grid project and how are they calculated? — Energy — U.S. Agency for International Development — usaid.gov. https://www.usaid.gov/energy/mini-grids/environment-health-safety/emissions, [Accessed 18-09-2024].

(6) Tarpeh, W. A.; Barazesh, J. M.; Cath, T. Y.; Nelson, K. L. Electrochemical stripping to recover nitrogen from source-separated urine. *Environmental science & technology* **2018**, *52*, 1453–1460.

(7) Liu, M. J.; Neo, B. S.; Tarpeh, W. A. Building an operational framework for selective nitrogen recovery via electrochemical stripping. *Water research* **2020**, *169*, 115226.

(8) Arredondo, M. R.; Kuntke, P.; Ter Heijne, A.; Hamelers, H. V.; Buisman, C. J. Load ratio determines the ammonia recovery and energy input of an electrochemical system. *Water research* **2017**, *111*, 330–337.

(9) Vecino, X.; Reig, M.; Gibert, O.; Valderrama, C.; Cortina, J. Integration of liquid-liquid membrane contactors and electrodialysis for ammonium recovery and concentration as a liquid fertilizer. *Chemosphere* **2020**, *245*, 125606.

(10) Li, Y.; Wang, R.; Shi, S.; Cao, H.; Yip, N. Y.; Lin, S. Bipolar membrane electrodialysis for ammonia recovery from synthetic urine: experiments, modeling, and performance analysis. *Environmental Science & Technology* **2021**, *55*, 14886–14896.

(11) Rodrigues, M.; De Mattos, T. T.; Sleutels, T.; Ter Heijne, A.; Hamelers, H. V.; Buisman, C. J.; Kuntke, P. Minimal bipolar membrane cell configuration for scaling up ammonium recovery. *ACS sustainable chemistry & engineering* **2020**, *8*, 17359–17367.

(12) Chen, W.; Grimberg, S.; Rogers, S.; Kim, T. Ammonia recovery from domestic wastewater using a proton-mediated redox couple. *ACS Sustainable Chemistry & Engineering* **2021**, *9*, 12699–12707.
